# Metronidazole Suspension for Paediatric Use in Developing Countries: Formulation, Quality, and Stability

**DOI:** 10.3390/pharmaceutics17060787

**Published:** 2025-06-17

**Authors:** Francesca Baratta, Chiara Zingarelli, Federica Fanton, Editson Lamy, Gaetano Di Lascio, Paola Brusa

**Affiliations:** 1 Department of Drug Science and Technology, University of Turin, Via Pietro Giuria 9, 10125 Turin, Italy; chiara.zingarelli@unito.it (C.Z.); federica.fanton@unito.it (F.F.); paola.brusa@unito.it (P.B.); 2 Aid Progress Pharmacist Agreement Non-Profit Association, Via Pietro Giuria 9, 10125 Turin, Italy; gaetano.dlascio@gmail.com; 3 Pharmacy Service, Foyer Saint Camille Hospital, 48 Rue Eben Ezer Marin 12, Route Nationale # 1, Varreux, Croix-des-Bouquets, Port-au-Prince HT6110, Haiti; leditson@gmail.com

**Keywords:** metronidazole suspension, paediatric formulae, compounding, pharmacy, developing countries

## Abstract

**Background/Objectives.** The paediatric population is a heterogenous group that is known to be a therapeutic orphan despite the recent incentives to promote the development of children’s formulations. Especially in low and middle-income countries, there is still a worldwide shortfall for the treatment and prevention of a variety of paediatric conditions. In this context, we developed a formulation specifically intended to administer metronidazole to paediatric patients using basic and low-cost excipients and with a simple set-up method. **Methods**. Various mixtures of excipients were prepared to obtain a suitable metronidazole liquid formulation at a concentration of 250 mg/5 mL. The best formula was tested for its quality and stability, assessing the uniformity of content, the pH, and the dispersion quality. We evaluated the stability of the preparation for 180 days at room temperature (25 +/− 2 °C), in a thermostatic oven (40 +/− 2 °C), and in a fridge (4 +/− 2 °C). **Results.** The tests performed gave excellent results. No variation greater than 10% was detected in the metronidazole concentration or in pH values after 180 days regardless of the temperature conditions during storage. Moreover, the microscope analysis confirmed the absence of significant differences over time. **Conclusions.** The results were consistent in different environmental conditions, ensuring the possibility of using the formulation even in those tropical countries where is not always possible to guarantee the conservation of medicines in controlled conditions. Moreover, the simple composition and easy preparation procedure make it possible to produce the suspension in any context, ensuring the quality of the finished product.

## 1. Introduction

The paediatric population is a heterogenous group, from new-born to adolescent, that may be affected by the same pathologies as adults and, consequently, require treatment with the same active molecules. However, these are often commercially unavailable in formulations that contain paediatric dosages. In addition, there is a lack of data that specify the efficacy and tolerability amongst children for industrial products approved for adult use [1,2]. The paucity of child-friendly formulations means that in the field of paediatric therapy, the practice of using off-label prescriptions is widespread, with the risk of administering non-optimal dosages, inefficacious therapies, or increasing the risk of adverse reactions [3,4]. Moreover, the excipients contained in these products may also be unsuitable for children, even in small quantities [5].

In recent years, there have been a number of initiatives worldwide that have attempted to foster and improve the development of paediatric medicines [4]. Although these initiatives have resulted in an increase in the number of paediatric medicines available, providing new directions for the use of approved medicines in the paediatric field [6,7], little attention has been given to improving the situation for older or off-patent medicines in formulations and dosages suitable for the paediatric population. The active ingredients in question account for most of the medicines listed as essential for children by the World Health Organisation (WHO), but only a third of the oral preparations are commercially available in formulations appropriate for children in the age group between one and five years old [8,9]. Despite regulatory incentives, there is still a worldwide shortfall for the treatment and prevention of a variety of conditions, especially in low- and middle-income countries [10].

Considering all the issues above regarding the availability of paediatric medicines, the prescription of extemporaneous preparations becomes a necessity and, thus, a common activity among paediatricians [5].

Oral liquid formulations are generally recommended for children: these are easily swallowed, and facilitate, in younger children, the adjustment of the dosage according to the body weight of the patient. However, there are also some disadvantages: the dosage and volume of the medicine may be limited by the solubility of the substance to be administered. Moreover, ensuring the stability of the formulation may require buffers, antioxidants, or preservatives [11,12]. Another factor, which must not be overlooked, is the palatability of the formulation. In the 1964 film *Mary Poppins* by Walt Disney, Mary sings “Just a spoonful of sugar makes the medicine go down.” Nobody could have imagined that this idea would be the subject of extensive research in the following decades to understand how the “sugar” helps to mask the bitter taste of medicines [13,14].

It is a fact that many active ingredients indeed have a bitter taste and this represents a potential hurdle to adherence among the paediatric population. Taste is of primary importance to children in deciding whether a food is acceptable or not; hence, this aspect must also be taken into consideration when developing medicines for the paediatric population: the sensory system matures gradually after birth and the responses of children to certain tastes differ greatly from those of an adult. Among these differences is the preference for sweet foods and a greater rejection of bitter tastes. Experimental studies have indicated that this preference for sweet tastes is a consequence of the basic biology of children [15,16,17].

Metronidazole is a synthetic nitroimidazole antibiotic with a particularly unpleasant taste. In developing countries (DCs), according to the WHO’s list of essential medicines for children [8], metronidazole is prevalently employed for the treatment of amoebic dysentery and giardiasis, infections that are associated with a high infant mortality and morbidity rate in DCs, as these are the cause of approximately 2.5 million deaths every year and have long-term effects on growth and cognitive function. Oral forms of metronidazole are commercially available but generally not in DCs, particularly in rural areas.

The *A.P.P.A.*^®^ Project [18,19,20,21] is an international health cooperation project whose main aim is to open galenic laboratories for the compounding of personalised medicines in DCs. The project was established at the University of Turin more than twenty years ago in collaboration with the non-profit association named Aid Progress Pharmacist Agreement. In the areas where the *A.P.P.A.^®^* project is active—currently Angola, Cameroun, Chad, Haiti, and Madagascar—the availability of medicines designed specifically for paediatric use is extremely scarce, and, in any case, the few available products are likely to be counterfeited. Therefore, it is of fundamental importance to enable the local health organisations to prepare their own medicines for the treatment of their patients, whether paediatric or adult, with high-quality, safe, and efficacious components.

In 2024, there was an outbreak of parasitic intestinal dysentery in the Haitian population (>30%) [22,23]. In this context, the aim of the present study was to develop an oral liquid metronidazole paediatric formulation using basic and economical excipients and employing a simple preparation procedure. Our goal is to obtain a formulation that is easy to prepare in the laboratories of the Haitian hospitals involved in the *A.P.P.A.^®^* Project and, more generally, in community pharmacies in low-income countries and that does not require temperature-controlled storage that make it difficult to store in a patient’s home.

## 2. Materials and Methods

### 2.1. Selection of Excipients

Various mixtures of excipients were prepared to obtain a suitable formulation: the principal criterion was its organoleptic properties. In order to make the formulation accessible even in low-income countries, the cost of raw materials was also taken into consideration. For the same reason, only raw materials whose storage instructions did not entail the need for temperature-controlled storage of the finished medicinal product were considered.

Due to the poor solubility of metronidazole in solvents, such as water, that are suitable for administration to paediatric patients, it was decided to develop a suspension. Thereafter, various combinations of sweeteners and flavours were prepared to make the formulation palatable.

In particular, the following ingredients were considered as sweeteners: erythritol, glucose, maltodextrin, sodium saccharin, sorbitol solution 70%, sucrose, sucrose syrup, and xylitol. Water-soluble flavours were also used: banana, lemon, orange, and strawberry. Solubilisers (ethanol, glycerol, propylene glycol, polysorbate 80), suspending agents (microcrystalline cellulose, sodium carboxymethyl cellulose), preservatives (sodium benzoate, sodium methyl p-hydroxybenzoate), and pH regulators (citric acid monohydrate, sodium citrate) were also tested.

The various compositions prepared to identify the most suitable formulation are reported in Table 1. The detailed quantity of each raw material is available as Supplementary Material.

The raw materials complied with the relevant monograph of the *European Pharmacopoeia* and were all purchased from a pharmaceutical supplier company (Farmalabor s.r.l, Canosa di Puglia, Bari, Italy).

### 2.2. Suspension Preparation

The best formula as regards organoleptic properties was prepared with metronidazole at a concentration of 250 mg/5 mL. The preparation procedure is as follows: weigh out the hydrophilic components (sodium methyl p-hydroxybenzoate, sodium saccharin, orange flavour, erythritol, and maltodextrin), add each component to purified water and mix until completely dissolution (mixture A). Mix metronidazole, sodium carboxymethylcellulose, and microcrystalline cellulose according to the progressive dilution procedure (mixture B). Add mixture A to mixture B and mix until the powders are completely suspended. All suspensions were prepared manually.

### 2.3. Suspension Quantitative Analysis

The evaluation of the uniformity of metronidazole content was carried out using a standard procedure designed in accordance with the directives set out in the *European Pharmacopeia* [24].

In particular, the quantity of metronidazole in the studied formulation batches was assessed by a high-performance liquid chromatography (HPLC) method. The instrument used was a YL9300 liquid chromatograph, Younglin Instruments Co., Anyang-si, Republic of Korea. It was equipped with an ultraviolet (UV) detector set at 230 nm. The column was the Agilent XDB-C18, 4.60 × 250 mm, 5 µm. The mobile phase was prepared as a mixture of methanol (20%) and water (80%). The flow rate was 1 mL/minute, the elution time was 6 min. The injection volume was 20 µL. The calibration curve of metronidazole was drawn using metronidazole analytical standard in methanol solution at concentrations between 0.020 and 0.330 mg/mL. The samples to be analysed were appropriately diluted with methanol to obtain a concentration within the range of the calibration curve. In particular, 980 µL of methanol was added to 20 µL of the suspension to be quantified. The sample was then vortexed (Velp Scientific ZX3 vortexer) for one minute and then filtered (Teknokroma nylon syringe filters 0.45 µm 13 mm ⌀ pk/100). The supernatant was appropriately diluted and analysed.

The concentration of metronidazole in the suspension samples was also evaluated by spectrophotometric assay between 190 and 600 nm. The instrument used was a V-730 UV-Visible Spectrophotometer, Jasko Inc., Tokyo, Japan. The calibration curve was made using metronidazole analytical standard in methanol ranging from 0.01 to 0.04 mg/mL. The samples to be analysed were appropriately diluted with methanol to obtain a concentration within the range of the calibration curve. In particular, 150 µL of each suspension lot to be analysed was added to 1 mL of methanol. The sample was then vortexed (Velp Scientific ZX3 vortex machine, Usmate Velate, Italy) for one minute and then centrifuged for 6 min at 6000 revolutions per minute (Beckman Coulter Microfuge 18, Cat model, Krefeld, Germany). The supernatant was then appropriately diluted and scanned. [25].

All chemicals were analytical grade (Merck KGaA, Darmstadt, Germany). Quantification experiments were performed at room temperature (15–25 °C).

### 2.4. Evaluation of Suspension Stability

The stability test for the metronidazole suspensions was conducted on nine lots (three production batches) of the selected formulation. Each lot was analysed after preparation to define the initial conditions and then subdivided into three samples: the first was stored at room temperature (25 +/− 2 °C), the second was stored in a thermostatic oven (40 +/− 2 °C), and the third was stored in a fridge (4 +/− 2 °C). Studies were conducted in conditions of relative humidity between 50 and 70%. The samples were analysed immediately after preparation and then every 30 days in each storage condition for a total period of six months. All of the samples were assessed for uniformity of content as described in Section 2.3, pH value (Hanna HI 9321 pH meter AC/DC input 110 V, Hanna Instruments Italia, Villafranca Padovana, Italy), and dispersion homogeneity.

In particular, the visual quality over time of the dispersion was assessed by an optical microscope (LEICA DM 2500 microscope, Leica Microsystems GmbH, Wetzlar, Ger-many) connected to a digital camera (630 magnification).

Moreover, it was also verified that each sample, even in case of phase separation as prescribed by the *European Pharmacopoeia* [24], could be redispersed by simple manual shaking.

Tests were repeated in triplicate. For each parameter, the mean of the results obtained and the standard deviation (SD) were calculated. The values were accepted for SD < 2%. All samples were stored away from light.

## 3. Results

To prepare a metronidazole suspension with a concentration of 250 mg/5 mL with organoleptic properties suitable for administering to paediatric patients, various formulations were prepared with the combinations of ingredients reported in Section 2.1. The formulation with the best organoleptic properties was sample no. 25, with the excipient combination reported in Table 2.

The selected formula was then tested for uniformity of content as described in Section 2.3 and for stability as described in Section 2.4.

The results obtained from the analysis of uniformity of content show that the concentration of metronidazole in each lot corresponds with the expected value. Both the chromatography and the spectrophotometric analyses reveal that, regardless of the temperature conditions during storage, the variation in concentration of metronidazole is less than 10% across all the lots.

The averages of the obtained results in the initial conditions and after 180 days are reported in Table 3 and in Table 4 for the analysis carried out using HPLC and spectrophotometry, respectively. The complete results are available as Supplementary Material.

Table 5 reports the average of the detected pH values: it is evident that no sample over time has undergone a significant change (<10%) in pH values.

**Figure 1:**
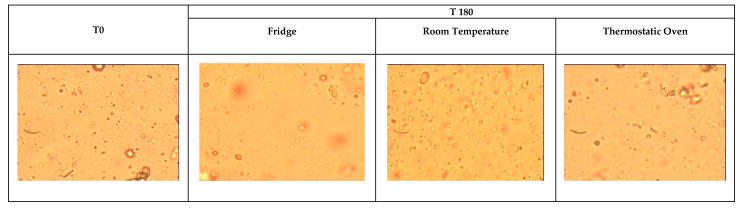
Microscope examination. T0: initial conditions; T180: 180 days of storage.

The microscope analysis (Figure 1) confirms the absence of any substantial difference in the tested suspensions, either in the initial conditions and throughout the period of the tests. The average diameter of the suspended particles was found to be less than 300 nm during the entire test period for all the tested samples. For all the tested suspensions, the separation phase was evident approximately one month on average after the date of preparation in any of the applied storage conditions. However, as indicated by the *Pharmacopoeia*, it was possible to manually redisperse the solid phase.

## 4. Discussion

The development of suitable paediatric formulations is certainly an issue of particular importance, in terms of both the scarcity of specific formulations for this age group and of liquid formulations.

Metronidazole may be administered intravenously, orally, or topically. For oral administration, it is commercially available in tablet, capsule, or suspension forms. In regards to suspensions, industrial medicines with concentrations of metronidazole or metronidazole benzoate ranging from 200 to 500 mg/5 mL are commercialised in some countries of the world. The administered dosage usually ranges between 35 and 50 mg/kg/day [26]. These preparations must be stored between 20 °C and 25 °C (brief exposure to 15 °C to 30 °C are usually permitted) [27,28,29]. The bitter metallic taste of metronidazole in its free base form in some commercial preparations has been masked by using the ester form, metronidazole benzoate, which has a blander taste. However, there are some questions over the bioavailability of the ester form that is, in any case, not widely available on the market as a raw material [13,30].

There are several factors that influence the choice of the antibiotic to be prescribed by a medical doctor, but the taste is not usually, or correctly, considered among these [31]. It is, therefore, fundamental to be able to treat the paediatric population with metronidazole in a suitable formulation.

In light of the above points, the study aimed to develop a pleasant-tasting oral liquid formulation for paediatric use in which the composition and preparation procedures are specifically designed for disadvantaged areas. Indeed, the suspension preparation method was designed to be very simple so as to be easily replicated in a compounding laboratory of any hospital or community pharmacy, even in DCs.

A variety of sweeteners and flavours were evaluated to identify the most suitable combination that was able to mask the taste of the metronidazole. The selection process for the formulation took into account their acceptable daily intake (ADI) and their potential side-effects [32,33,34]. The optimum sweetener system was identified in a combination of maltodextrin, erythritol, and sodium saccharin. Sodium saccharin, which for a long time had an ADI of 5 mg/kg/day in the general population, or 2.5 mg/kg/day in the paediatric population according to the literature, was recently revised in terms of its safety for food use. This revision led to a substantial increase in the safe daily dose to 9 mg/kg/day for the European Food Safety Authority (EFSA) in food [35,36]. This process confirmed the safe use of this sweetener in the developed formulation: the maximum dose foreseen for therapies with metronidazole totals a daily intake of 1.9 mg/kg/day of sodium saccharin, already consistent with the more restrictive indications above reported.

Polyalcohols such as erythritol are generally considered safe and are classified by the Food and Drug Administration (FDA) as generally recognised as safe (GRAS) [37]. In addition, children are not usually as sensitive as adults to the gastrointestinal effects of erythritol as adults [38]. In 2023, the EFSA established an ADI of 0.5 g/kg/day for erythritol as a safeguard against its immediate laxative effects, but also any adverse long-term effects secondary to diarrhoea, such as electrolyte imbalance [39] This ADI value is five times higher than the maximum dose of erythritol, which it is possible to assume in our suspension. Maltodextrin, which the FDA considers as a GRAS component [37], has no ADI for the ESFA, and, in general, any product for children containing maltodextrin is considered safe [40]. Orange flavour was added to the sweetening system as this is generally well accepted by the paediatric population.

Regarding the microbiological stability of the formulation, a key factor especially for DCs, it is important to underline that, according to the European Medicines Agency (EMA), the use of preservatives is deemed appropriate in multi-dose formulations but for many preservatives, only limited data are available regarding the maximum safe dose for children. The addition of such components would, therefore, be limited to the lowest quantity possible [41]. Parabens, a class of compounds including sodium nipagin (sodium methyl p-hydroxybenzoate) used in the formulation, are considered the safest class of compounds for paediatric use [35]. Parabens are usually used in concentrations ranging from 0.01% to 0.2% and the recommended maximum daily dose is 10 mg/kg/day [42]. This concentration is significantly higher than the one used in our study.

For oral liquid medicines, the EMA recommends that the administered volume should not more than 5 mL in children less than 5 years of age [41]. This indication considers the fact that lower volumes can generally be administered more easily and are more tolerable for children; hence, the developed formula adheres to this recommendation.

Once the composition of the formulation had been defined, it was fundamental to assess its stability. Therefore, tests were carried out in different environmental conditions: the tests performed at room temperature were applied to define the stability in the storage conditions generally foreseen for medicinal products. Stability tests performed at higher temperatures had two purposes: first, to reduce the duration of the analysis time, considering that one month’s storage in these conditions is the equivalent of four months storage in standard conditions [20]; secondly, the applied temperatures help to predict stability in environments, such as those of DCs, where it is often impossible to ensure storage at temperatures below 25 °C. Finally, the tests carried out on samples stored in a fridge verify the stability of the product in these conditions if essential.

Regarding the quantification of metronidazole to evaluate the uniformity of content as well as the stability of the formulation, two analytical techniques were employed: chromatography and spectrophotometry. The scientific literature does not usually consider spectrophotometry as a quantitative method for active ingredients [25]. However, and in light of the fact that the *European Pharmacopoeia* does not prescribe a specific analytical method to quantify the concentration of active ingredients in a medicine (the *European Pharmacopoeia* states that “a suitable method” must be used) [24], we have also applied spectrophotometry as a quantitative method. The reasoning behind this choice is the need to develop a quantification method that can be performed using low-cost, easy-to-use instrumentation. The end is to ensure that the method, once its efficacy has been assured, can be used even in disadvantaged areas. In this light, the results obtained from the present study are certainly positive, because the data obtained from HPLC and spectrophotometry are substantially analogous.

## 5. Conclusions

The low availability of commercially oral paediatric formulations based on metronidazole inevitably means that many times, the therapy for this group must be prepared as a galenic formulation. Hence, we chose to focus our research on the development of a suspension with simple ingredients to make the preparation method feasible in any context using the equipment commonly available in any local or hospital galenic laboratory, but, at the same time, able to assure the quality of the finished product. Therefore, it was not only important but necessary to develop standard operating procedures that rely on low-cost instrumentation to assess on-site both the quality and the stability of the suspension.

## Figures and Tables

**Table 1:** Excipient mixtures tested for metronidazole suspension.

Ingredients	1	2	3	4	5	6	7	8	9	10	11	12	13	14	15	16	17	18	19	20	21	22	23	24	25	26	27	28	29	30	31	32	33
Banana flavour (water-soluble)			x					x																									
Citric acid monohydrate																															x		x
Purified water	x	x	x	x	x	x	x	x	x	x	x	x	x	x	x	x	x	x	x	x	x	x	x	x	x	x	x	x	x	x	x	x	x
Erythritol									x	x	x						x		x	x			x		x	x		x		x	x		
Ethanol																																	x
Glucose																					x	x	x	x		x							
Glycerol																					x	x	x	x		x							
Lemon flavour (water-soluble)	x				x		x		x	x		x		x		x	x	x	x	x	x	x	x	x		x			x			x	x
Maltodextrin	x					x						x	x					x	x					x	x	x	x	x		x	x		
Microcrystalline cellulose	x	x	x	x	x	x	x	x	x	x	x	x	x	x	x	x	x	x	x	x	x	x	x	x	x	x	x	x	x	x	x	x	
Orange flavour (water-soluble)				x		x					x										x	x	x	x	x	x	x	x	x	x	x	x	
Propylene glycol																					x	x	x	x		x							
Sodium benzoate																											x	x	x	x	x		
Sodium carboxymethyl cellulose	x	x	x	x	x	x	x	x	x	x	x	x	x	x	x	x	x	x	x	x	x	x	x	x	x	x	x	x	x	x	x	x	
Sodium citrate																															x		x
Sodium methyl p-hydroxybenzoate	x	x	x	x	x	x	x	x	x	x	x	x	x	x	x	x	x	x	x	x	x	x	x	x	x	x						x	
Sodium saccharin												x	x	x	x	x	x	x	x	x	x	x	x	x	x	x	x	x	x	x	x		x
Sorbitol solution 70%																					x	x	x	x		x							
Strawberry flavour (water-soluble)		x											x		x																		
Sucrose					x																x												
Sucrose syrup	x	x	x	x	x	x	x	x	x	x	x	x	x	x	x	x	x	x	x	x									x			x	x
Polysorbate 80																					x	x	x	x		x	x		x	x	x		
Xylitol						x	x	x		x	x			x	x	x	x	x		x		x							x				

**Table 2:** Suspension composition for 100 mL.

Ingredients	Amount (g)
Metronidazole	5.00
Orange flavour (water-soluble)	4.72
Sodium carboxymethyl cellulose	1.00
Microcrystalline cellulose	5.00
Erythritol	9.40
Maltodextrin	9.40
Sodium methyl p-hydroxybenzoate	0.10
Sodium saccharin	0.19
Purified water	95.60

**Table 3:** HPLC results.

		T0		T 180					
				Room Temperature		Thermostatic Oven		Fridge	
		Mean Area Values	Average	Mean Area Values	Δ % Compared to Average Value at T0	Mean Area Values	Δ % Compared to Average Value at T0	Mean Area Values	Δ % Compared to Average Value at T0
**Batches**	**A**	2429	2378	2343	−1.5%	2286	−3.8%	2535	6.6%
	**B**	2411		2387	0.4%	2264	−4.8%	2240	−5.8%
	**C**	2293		2189	−7.9%	2396	0.8%	2270	−4.5%

T0: initial conditions; T180: 180 days of storage.

**Table 4:** Spectrophotometric results.

		T0		T 180					
				Room Temperature		Thermostatic Oven		Fridge	
		Mean Absorbance Values	Average	Mean Absorbance Values	Δ % Compared to Average Value at T0	Mean Absorbance Values	Δ % Compared to Average Value at T0	Mean Absorbance Values	Δ % Compared to Average Value at T0
**Batches**	**A**	2.2171	2.2076	2.2744	3.02%	2.1736	−1.54%	2.2590	2.33%
	**B**	2.2449		2.3669	7.21%	2.2552	2.15%	2.0151	−8.72%
	**C**	2.1609		2.2994	4.16%	2.3295	5.52%	2.0817	−5.70%

T0: initial conditions; T180: 180 days of storage.

**Table 5:** pH values.

		T0		T 180					
				Room Temperature		Thermostatic Oven		Fridge	
		Mean pH Value	Average	Mean pH Value	Δ % Compared to Average Value at T0	Mean pH Value	Δ % Compared to Average Value at T0	Mean pH Value	Δ % Compared to Average Value at T0
**Batches**	**A**	8.41	8.35	7.88	−5.59%	8.36	0.16%	8.29	−0.68%
	**B**	8.16		7.83	−6.19%	8.30	−0.56%	8.70	4.23%
	**C**	8.47		7.96	−4.63%	8.26	−1.04%	7.80	−6.55%

T0: initial conditions; T180: 180 days of storage.

## Data Availability

Data is contained within the article.

## Supplementary Materials

The following supporting information can be downloaded at: https://www.mdpi.com/article/10.3390/pharmaceutics17060787/s1.

## Abbreviations

*A.P.P.A.* ^®^	Aid Progress Pharmacist Agreement
ADI	Acceptable Daily Intake
DCs	Developing Countries
EFSA	European Food Safety Authority
FDA	Food and Drug Administration
GRAS	Generally Recognised As Safe
HPLC	High-Performance Liquid Chromatography
SD	Standard Deviation
T0	Initial conditions
T180	180 days of storage
UV	Ultraviolet
WHO	World Health Organisation
